# Heavy metals in biological samples of cancer patients: a systematic literature review

**DOI:** 10.1007/s10534-024-00583-4

**Published:** 2024-02-12

**Authors:** Donatella Coradduzza, Antonella Congiargiu, Emanuela Azara, Ismaeil Mohammed Abulkahar Mammani, Maria Rosaria De Miglio, Angelo Zinellu, Ciriaco Carru, Serenella Medici

**Affiliations:** 1 Department of Biomedical Sciences, University of Sassari, Viale San Pietro 43/B, 07100 Sassari, Italy; 2 Institute of Biomolecular Chemistry, National Research Council, Sassari, Italy; 3 College of Health Sciences, University of Duhok, Duhok, Iraq; 4 Department of Medical, Surgery and Experimental Sciences, University of Sassari, Sassari, Italy; 5 Department of Chemistry and Pharmacy, University of Sassari, Vienna 2, 07100 Sassari, Italy

**Keywords:** Heavy metals, Trace elements, Breast cancer, Lung cancer, Gastric cancer, Prostate cancer

## Abstract

The majority of the so-called heavy metals are suspected to be involved in a number of pathologies and play a role in human carcinogenesis. Some of them (i.e. arsenic (As), cadmium (Cd), chromium (Cr), lead (Pb), mercury (Hg) and nickel (Ni)) have been defined as carcinogens, increasing the susceptibility of tumor development and progression in humans. Moreover, Ni, Cr, Cd, Hg, and Pb together with zinc (Zn) and iron (Fe), may be capable of stimulating the progression of breast cancer and reducing a patient’s sensitivity to treatment through alterations to DNA methylation. In patients with gastric cancers, levels of various heavy metals are augmented and hypothesized to amplify the expression of the human epidermal growth factor receptor type 2 gene. Cd may increase the risk of lung cancer development and have a negative impact on the overall survival of lung cancer patients. To investigate the relation between heavy metals in biological samples and risk, occurrence and survival cancer individuals, a comprehensive review work was performed, with a focus on breast, lung, prostate and gastric cancers. An extensive search strategy was devised to ensure relevant literature could be identified, with the PECO framework being adopted to facilitate this and identify key search terms. As evidenced in this review, there is substantial data to support the hypothesis that heavy metals influence tumor development and progression. Unluckily the number of papers dealing with the determination of metals directly in samples from cancer tissues is still rather limited, so we decided to expand the scope of this review also to analyses carried out on other biological samples, as urine, plasma, hair, nail, etc. The studies reviewed showed that several limitations and current knowledge gaps are present in the literature that require further investigation to improve our comprehension of the impact of different heavy metals on tumorigenesis.

**Graphical abstract:**

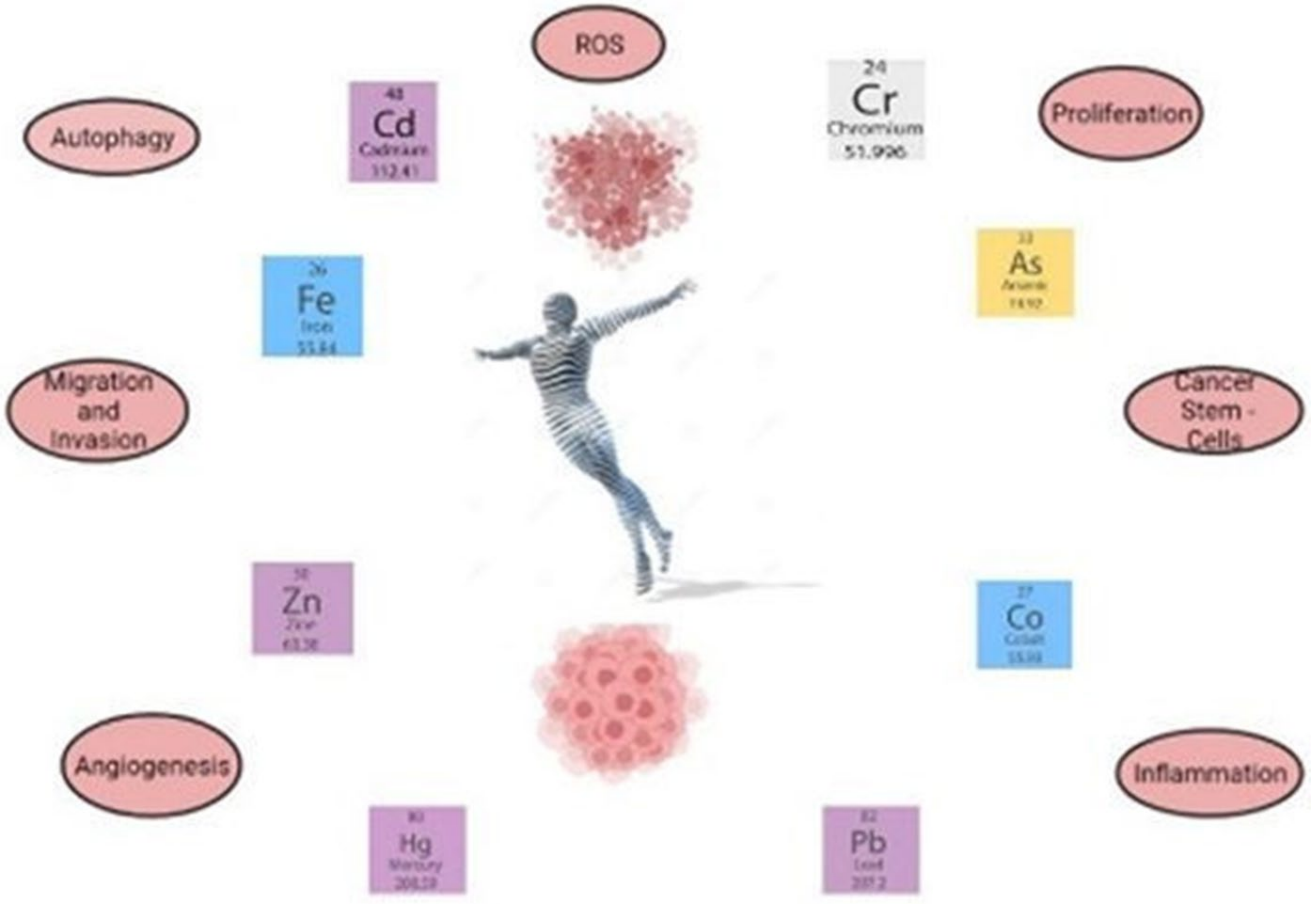

## Introduction

Metals play many roles in the body, according to their nature. Some of them are the so called “essential” metals, belonging to the first (alkali) and second (alkaline earth) groups: sodium (Na) and potassium (K) are mainly charge carries; magnesium (Mg) and calcium (Ca) participate to the assembly of hard structures via bio-mineralization (teeth, bones etc.) and have structural functions in macromolecules (proteins, DNA); light, first-row transition metals, defined as “essential in traces” like iron (Fe), copper (Cu), or zinc (Zn), participate in prosthetic groups of proteins or enzymatic cofactors, with the redox-active ones busy in transferring electrons and catalyzing enzymatic redox reaction [1]. On the contrary, heavy metals have no biological function, and exert a toxic action on the body already at low concentrations. Even essential metals, in particular Fe, Zn, Cu, and to a lesser extent also Cr (in its + 3-oxidation state), can be noxious, when their amount in the organism exceeds the threshold limits: as Paracelsus said, “the dose makes the poison”.

Metal toxicity has long been recognized and studied for its consequences on human health. Non-essential metals such as Cd, Hg, Ni, and Pb, can be harmful mainly, but not only, because they interfere with metabolic processes by substituting essential divalent cations (e.g. Ca, Mg, Fe and Zn) in enzymes, proteins and hard structures, such as bones or teeth. Trace elements can be noxious to the human organism when their concentration exceeds precise physiological limits, and their toxicity is particularly related to their redox activity (except for Zn), which can induce oxidative stress, ROS production etc., in a Fenton (Fe) or Fenton-like (Cu, Cr, etc.) chain of reactions [1]. The cellular damage caused by heavy metals is linked to different characteristic of these elements, such as solubility, oxidation states, hard-soft character, binding attitudes, presence of different forms, which can influence their speciation in biological systems.

The speciation of an element is the chemical form(s) that it takes in solution, in this case biological fluids. Except for the nanoparticles of some noble elements (e.g. Ag, Au, Pt), metals in the body are never found in their metallic form, but as cations, bound in salts (e.g. sulfides, phosphates, etc.), oxides or complexes, and this is what we will refer to, when generically speaking of metals. Biotransformation mechanisms are able to convert inorganic forms of different cations into organometallic species, for instance through methylation processes [2]. Heavy metals can interact and make complexes with biological molecules such as DNA, enzymes, proteins and peptides (e.g. albumins, metallothioneins, glutathione, etc.) by binding to sulfur (e.g. cysteine) or nitrogen (e.g. histidine) groups, and this is the way in which they are generally found in cells and tissues. Free metal ions (aquo complexes) are infrequently found, and this is their most active and toxic form [1, 3–5].

Metal exposition is not a rare event for humans. Metals are a core component of Earth and its crust and are present in every aspect of the environment [6]. Besides, heavy metals have multiple applications in industrial, domestic, agricultural, medical, and technological industries, causing concerns to be raised about their potential impact on human health [7, 8]. Collectively, this has triggered the absorption of such heavy elements in humans, primarily from crops, water, vegetables, and sediments [9, 10]. Although not widely comprehended, the toxicity induced by the accumulation of heavy metals has been established [11]. As just reminded, in the human body, these metallic elements can impair metabolism, cause oxidative stress, and result in the development of numerous diseases and conditions [12, 13]. For instance, Hg is able to induce neurological disorders and damage to the central nervous system (e.g. the mad hatter syndrome, also known as erethismus mercurialis [14], and the so-called Minamata disease, from the Hg poisoning among people from Minamata bay, in Japan [15]; Pb can lead to deficiencies in cognitive development in children [16] or to saturnism, a condition already known by the Romans, together with a series of other disfunctions such as anemia and neurological, respiratory, urinary, and cardiovascular disorders [17]; Cr(VI) causes oxidative damage that can lead to hemolysis and, ultimately, kidney and liver failure, but also gastrointestinal and skin disorders [18].

Cancer Research institution (UK) reported an estimated 18.1 million new cancer cases worldwide in 2020, with 10 million deaths, of which 33% were caused by smoke [19]. In a similar way the National Center for Health Statistics reported that, in 2019, there were 1,918,030 new cancer cases and 609,360 cancer death in the United States [20]. This includes an estimated 350 deaths per day from lung cancer, one of the most prevalent malignancies and causes of cancer death. Long-term exposure to some heavy metals and their compounds has been linked to the development of cancer and tumor formation. For example, Cd is proposed to mimic estrogenic effects, as a result of its ability to form a high-affinity complex with the hormone binding domain of the estrogen receptor, promoting the development of breast cancer [21].The accumulation of Cd has also been found to increase the risk of lung cancer occurrence, with high exposure to this heavy metal resulting in poor prognosis in patients with lung cancer [22]. Additionally, numerous heavy metals, including Cr, Ni, and Cd, are proposed to destroy the gastric mucosa barrier, triggering inflammation and tissue damage, and leading to gastric cancers [23]. The International Agency for Research on Cancer has classified As, Cd, Cr and Ni as group one carcinogens [24–26]. These elements and their compounds induce oxidative stress, DNA damage, and cell death processes, augmenting the risk of cancer and cancer-related conditions such as high blood pressure, arthritis, diabetes, lung disease, heart disease, or kidney disease [27]. Collectively, these metals represent a profound environmental risk factor for the development of several malignancies [28]. In patients with malignancies, the possible noxious role of heavy metals can be reflected in the biomarkers present in blood, tissues, skin, and nails, such as those related to ROS production, as evidenced in studies carried out on several species, from humans to aquatic moss [29–31]. This review aims to investigate the relation between heavy metals in biological samples and occurrence and survival cancer individuals, by summarizing the most recent evidence on the implications of repeated heavy metal exposure as an environmental risk factor for cancer development and progression, together with related evidences in other species and organisms that can be relevant for the discussion. The malignancies of focus for the purpose of this review are lung cancer, breast cancer, prostate cancer, and gastric cancer.

## Results and discussion

The results of the search strategy identified four prevalent malignancies in the current literature that are influenced by the presence of heavy metals in biological samples. The results and discussion of this review will, therefore, comprise research on heavy metals in the biological samples of lung, breast, prostate and gastric cancer patients.

### Lung cancer

Lung cancer is a prevalent malignancy that can be attributed to many environmental and genetic risk factors, particularly smoking [32] (Table 1). However, the role of prolonged heavy metal exposure as an environmental factor has been recently established [26, 31]. A 2022 study by Lee et al. carried out on urine samples from population residing close to a petrochemical industrial area with high air Cd concentration noted that air exposure to this metal increases the risk of lung cancer development and has a negative impact on the overall survival of lung cancer patients [22]. It has also been suggested that a relationship exists between the levels of soil contamination by heavy metals and lung cancer incidence [33, 34]. Tran et al. investigated the prevalence of Cd, As, Hg, and Pb in postsurgical tissues from individuals with and without non-small cell lung cancer (NSCLC). Two groups of biological samples were explored. In the first group, tissue samples from 15 patient cases with stage I and stage II NSCLC who underwent surgical resection were assessed. The findings indicate that the amount of Pb and Hg observed was not significant. On the other hand, Cd was the most prevalent heavy metal identified, and As was observed in moderate amounts. In the second group, 28 patients with a confirmed NSCLC, including adenocarcinoma, squamous cell carcinoma, or large cell carcinoma, and nine noncancer benign lung tissue samples were evaluated. In all tissue samples obtained from NSCLC patients, Cd was observed, and its levels were significantly higher in noncancer benign lung samples than NSCLC patients. Additionally, As was observed in moderate amounts in NSCLC patients, whilst both Hg and Pb amounts were negligible. These findings warrant the hypothesis that Cd and As may have an impact on the development of NSCLC; however, further studies are necessitated to corroborate these results [34, 35]. The presence of heavy metals in biological samples from lung cancer patients was reinforced by Pietrzak et al., who assessed the blood concentrations of As, Cd, Hg, and Pb in 336 patients with lung cancer. The relationship between these concentrations and overall survival was also reported. It was observed that Cd was the only heavy metal that significantly influenced overall survival in these patients. The findings also revealed that low blood.

Cd levels, defined as < 1.47 µg/L, may result in improved overall survival in treated patients with stage IA disease [36]. Although not widely investigated, it has also been suggested that serum levels of heavy metals may influence the development and progression of lung cancer. 440 patients were investigated by Bai et al. for the presence of 11 metals (Cu, Zn, Fe, Co, Mn, Mo, Rb, Se, Sn, Sr and V) in their blood samples, but only Zn seemed related to lung cancer, as high plasma levels of this metal were associated with lower incident risk of lung cancer, probably due to Zn slowing down telomere attrition and regulating the expressions of cancer-related genes [37]. Cu was, for a long time, considered as an active site metabolic cofactor solely [38], but lately it started to appear as an essential nutrient for tumour growth and metastasis that have an increased requirement of this metal, and the group of C. Chang has recently introduced the term “cuproplasia”, within the wider frame of “metalloplasias”, to indicate copper-dependent cell proliferation, akin to ferroptosis as an iron-dependent form of cell death [39]. In animal models (*Drosophila melanogaster* and mouse), Cu emerged in the evidence that is a dynamic signaling metal and metalloallosteric regulator, as for copper-dependent phosphodiesterase 3B (PDE3B) in lipolysis [40], mitogen-activated protein kinase 1 (MEK1) and mitogen-activated pro tein kinase 2 (MEK2) in cell growth and proliferation and the unc-51 like kinase 1 (ULK1) and unc-51 like kinase 2 (ULK2) in autophagy [41, 42]. Within the frame of the aforementioned cuproplasia, cuproptosis was investigated as novel copper-dependent cell death (in analogy with ferroptosis) to evaluate the association between single-nucleotide polymorphisms in cuproptosis-related genes and lung cancer risk. Some of the minor alleles examined correlated with an increased risk of lung cancer, while some others were associated with lower risk, evidencing a relation between cuproptosis-related genes an lung cancer risk [43]. Cobanoglu et al. observed that Zn may behave as a protective agent against lung cancer, whilst low Zn levels may facilitate the pathogenesis of tumor development [44]. Also, dysregulation of iron metabolic proteins is closely associated with the initiation and development of lung cancer [45]. The serum, bronchoalveolar lavage fluid, of patients with lung cancer contain elevated ferritin levels [46–48]; moreover lung cancer cells could increase iron intake by enhancing the effects of the transferrin protein (TF) and transferrin receptor (TfR) [48, 49]. Different authors boldly guess than an increase in Fe intake by lung cancer cells could be related to ferroptosis, one of the potential forms of death for lung cancer cells [50–52].

**Table 1:** Heavy metals and lung cancer

Metals	Cancer site	Sample size	Material	Level of metals	Impact	References
V, Cr, Mn, Ni, Cu, As, Sr, Cd, Hg, Tl, and Pb	Lung cancer	59 Patients with lung cancer 20 Patients with other malignancy 2338 General population	urine	Higher level of Cd	Increases risk of developing cancer; negative impact on the overall survival	Lee et al. (2022) [15]
Cd, As, Hg, and Pb	NSCLC	15 Patients with stage I and Stage II NSCLC 28 Patients with adenocarcinoma, squamous cell carcinoma, or large cell carcinoma 9 Noncancer benign lung tissue	postsurgical tissues	Cd and As levels were significantly higher.	Increase risk of developing NSCLC	Tran et al. (2014) [24]
Cd, As, Hg, and Pb	Lung cancer	336 Patients with lung cancer	blood	Higher level of Cd	Significantly influenced overall survival	Pietrzak et al. (2021) [25]
Zn, Cu, Pb, Zn, Fe, Co, Cd, Mn, Mg	Lung cancer	30 Lung cancer 20 Healthy controls	serum	Lower Zn levels may facilitate the tumor development	May facilitate the tumor development	Cobanoglu et al. (2010) [31]

### Breast cancer

Trace elements and heavy metals have been widely observed in biological samples of breast cancer patients and have been the focus of several research investigations over the past two decades [53–56] (Table 2). It has been evidenced that through multiple pathogenetic links, heavy metals are capable of stimulating the progression of breast cancer and reducing a patient’s sensitivity to treatment [57–59]. A prospective study by Cihan et al. analyzed the hair levels of 36 elements in 52 patients with stage III breast cancer, comparing them to those of healthy individuals. There were notable differences between the two patient groups; a higher level of Fe was observed in cancer patients, whilst the control participants had higher levels of Ca. The primary difference observed between the two groups concerned increased levels of heavy metals in the hair samples of cancer patients [32]. Likewise, Ionescu et al. observed a pathological accumulation of transition metals in breast tissue. This study analyzed the concentration of transition metals in 20 breast cancer biopsies, comparing the findings to the levels observed in eight healthy biopsies. Significantly higher levels of Fe, Ni, Cr, Zn, Cd, Hg, and Pb were observed in the cancer biopsies compared to the healthy ones. Hence, it was concluded that these metals might be involved in the malignant growth process [60]. Li studied the association between plasma heavy metals and the metabolome in patients with breast cancer, and the association with cancer development. In these patients, Cd was significantly ~ 15-fold higher in the plasma of patients with breast cancer compared with that in the control population, but also Cr, As and Pb were elevated by ~ 3.24, 2.14 and 1.52-fold, respectively. Moreover, small molecules, including amino acids and salts, were altered in the plasma of patients with breast cancer compared with the control population. Multivariate analysis has suggested that exposure to heavy metals, including Cd, As, Cr and Pb, may influence blood lipid levels and other small molecule metabolites, which in turn may be involved in breast cancer development [61].

A similar study, but on urine, investigated the correlation between heavy metals detected in urine and the urine metabolome of patients with breast cancer, and their association with cancer development [61]. As in plasma samples, the results demonstrated that Cd, Cr and As, were markedly increased in the urine of patients with breast cancer compared with the control population. Moreover, small molecule metabolites were altered in the urine of patients with breast cancer and the results were very similar to previous reports, indicating that environmental exposure to Cd, As, or Cr could be involved in breast cancer development [61]. O’Brien et al. have studied toenail-based metal concentrations in relation to young-onset breast cancer (diagnosis age < 50 years) of 1,217 disease-discordant sister pairs in the US-based Sister and Two Sister. They studied cadmium as a metalloestrogen, together with 9 other metals, such as As, Co, Cr, Cu, Mo, Pb, Sn, and V. Cd was associated with a small increase in risk, with no evidence of a dose-response relationship. In case-control studies, no association was observed between young-onset breast cancer and toenail concentrations of any metal evaluated [62]. A case-control study within the ORDET cohort by Pala et al. has observed that Cu is high and Zn low in blood and urine of women in 496 breast cancer cases compared with controls; in addition, increased Cu/Zn ratio in plasma and urine may both be an early marker of, and a risk factor for, breast cancer development [63]. The role of heavy metals in the air and the risk of postmenopausal breast cancer have also been discussed. In a study evaluating the association between air toxics, including heavy metals, and breast density, increased levels of Hg, Cd and Pb were found to be associated with postmenopausal breast cancer. The odds ratio was 1.1 in a cohort comprising 2587 breast cancer cases [64]. The rationale for this increase may lie with the hypothesized role of Cd and Ni as metalloestrogens, as a result of their ability to form high-affinity complexes with the hormone binding domain of the estrogen receptor thus mimicking the action of estrogens [21, 58].

Systematic Reviews and Meta-analyses have studied the association between heavy metals and breast cancer and reviewed the potential mechanisms systematically [65, 66]. 36 studies in different biological samples, comprising 4151 individuals from five continents around the world were included. It was found that especially in plasma and serum, Cu, Cd, and Pb concentrations were higher in patients with breast cancer, while Zn (in hair) and Mn concentrations were lower [65, 67]. Another systematic review and meta-analysis evaluated the associations between Fe and breast carcinogenesis. 23 studies were included assessing iron intake and/or biomarkers of iron status in relation to breast cancer risk, and dose-response meta-analyses were also performed to investigate linear and nonlinear associations. Heme iron intake was significantly associated with increased breast cancer risk, whereas no associations were found for dietary Fe, data that may have an impact and public health implications given the widespread consumption of (heme) iron-rich foods [68].

**Table 2:** Heavy metals and breast cancer

Metals	Cancer site	Simple size	Material	Level of metals	Impact	References
36 elements Ag, Al, As, Au, B, Ba, Be, Bi, Ca, Cd, Ce, Co, Cr, Cs, Cu, Fe, Ga, Hg, K, Li, Mg, Mn, Na, Ni, Pb, Pd, Rb, Rh, Sb, Sc, Se, Sn, Sr, Ti, V, Zn	Breast cancer (stage III)	52 Patients 52 Healthy controls	Hair	Cancer patients had higher levels of 18 elements: Al, As, B, Cd, Ce, Co, Cr, Cs, Cu, Fe, K, Li, Mn, Na, Rb, Rh, Sb, and V vs. healthy controls	These elements might be involved in development and progression of breast cancer	Cihan et al. (2011) [41]
Cd, Zn, Hg, Ni, Cr, Fe	Breast cancer	20 Patients 8 Healthy controls	Biopsies	Higher levels of Fe, Ni, Cr, Zn, Cd, Hg, and Pb	These metals might be involved in the malignant growth process	Ionescu et al. (2006) [47]
Cd, Cr, As, Pb, Cu	Breast cancer	105 Patients 35 Healthy controls	Plasma	Cd was ~ 15-fold higher. Cr ~ 3.24; As ~ 2.14; Pb ~ 1.52-fold higher.	May influence blood lipid levels and other small molecule metabolites, involved in breast cancer development	Li et al. (2020) [48]
Cd, Cr, As, Pb and Hg	Breast cancer	106 Patients 36 Healthy controls	Urine	Cd, Cr, As, was increased	Environmental exposure to Cd, As, or Cr could be involved in breast cancer development	Men et al. (2020) [49]
Zn, Cr, Co, Fe, Ni, Mn, Cd, Pb	Breast cancer	991 Patients 1223 Healthy controls	Different biological samples	In plasma and serum, Cu, Cd, and Pb concentrations were higher instead Zn, in hair, and Mg concentrations were lower	Higher Cd may be associated with the BC risk	Liu et al. (2022) [53]
Fe	Breast cancer	8449 Patients 11092 Healthy controls	/Plasma serum	–	Heme iron intake was significantly associated with increased breast cancer risk	Chang et al. (2019) [55]
Cu, Zn	Breast cancer	496 Patients	Blood and urine	High levels of Cu low levels of Zn	Increased Cu/Zn ratio in plasma and urine may be both an early marker of, and a risk factor for breast cancer development	Pala et al. (2022) [51]
Cd, Ni	Postmenopausal breast cancer	–	Tissue, urine, blood, hair	High levels of Cd and Ni	Cadmium and nickel have been hypothesized to play a role in breast cancer development by acting as metalloestrogens	Aquino et al. (2012) [45]

### Prostate cancer

Prostate cancer represents one of the most prevalent malignancies in men, particularly in industrialized countries [69]. Several heavy metals such as Cd, As, Zn and Fe have been indicated to play a role in the various biochemical processes underpinning the pathogenesis of this disease [70] (Table 3). This includes disruption of protection against oxidative damage in cellular respiration, genomic stability, immunity, apoptosis, and cell signaling [71, 72]. A 2019 study by Lim conducted a multiple metal analysis to explore the association between serum heavy metals and the occurrence of prostate cancer. A total of 141 cases were included in this study and were matched to 114 controls, with the concentrations of ten heavy metals being determined. The results identified four heavy metals, specifically As, Zn, Mn, and Sb that were significantly and positively associated with prostate cancer occurrence. Additional analyses revealed As and Zn to have the most significant impact on the risk of prostate cancer when all other metals were held fixed [73]. Likewise, Sarafanov et al. found that there is an association between Zn and Fe prostate tissue levels and the development of prostate cancer, since patients with biochemical (PSA) recurrence of disease showed 12% lower median Fe (95 µg/g vs. 111 µg/g) and 21% lower Zn (279 µg/g vs. 346 µg/g) concentrations in the normal-appearing tissue immediately adjacent to cancer areas [74]. Wu’s group conducted a study to investigate whether there was a relationship between heavy metal exposure and prostate-specific antigen (PSA). It was examined whether men without prostate cancer, aged ≥ 40 years, identified by the National Health and Nutrition Examination Survey 2003–2010, had a correlation between levels of total urinary As as urinary dimethylarsonic acid, blood Cd, blood Pb and total blood Hg levels and elevated PSA levels. It was found that men with elevated PSA had higher blood Cd and blood Pb levels than men with normal PSA. This significant relationship disappeared after adjusting for age, race/ethnicity, body mass index, smoking and education [51]. Besides heavy metal levels, associations have also been found between trace elements and heavy metal concentrations in biological samples of those with prostate cancer. A 2012 study by Karimi explored the relationship between heavy metals including Se, Zn, Cu, Mn, and Fe with prostate cancer [75]. A case-control study analyzing hair and nail samples from 100 patients revealed significantly lower mean levels of Se and Zn compared to controls. Conversely, elevated levels were observed for Fe and Mn [75]. Neslund-Dudas et al. described that Cd exposure from smoking may play a role in disease progression. Metal concentrations were measured for biochemical and distant disease recurrence in neoplastic tissues in paraffin. It was found that smokers had significantly higher Cd and lower Zn in non-neoplastic tissue than non-smokers [70].

**Table 3:** Heavy metals and prostate cancer

Metals	Cancer site	Sample size	Material	Level of metals	Impact	References
Mn, Cu, Zn, As, Se, Sb, Co, Cu, Cd and Pb	Prostate cancer	141 Patients 114 Healthy controls	Serum	Mn, Ni, Zn, As, Se and Sb had significantly higher concentrations among cases than controls	As, Zn have the most significant impact on the risk of prostate cancer	Lim et al. (2019) [59]
Cd, Fe, Zn, and Se	Prostate cancer	40 Patients cancerous tissue and healthy tissue	Tissue	Altered levels of Fe, Cd and Zn. Lower levels of Fe and Zn and higher levels of Cd in cancerous tissue samples	The complex interaction between metal homeostasis and cancer progression highlights the potential importance of these elements in the context of both cancer development and recurrence.	Sarafanov et al. (2011) [65]
Hg, As, Cd, Pb	Prostate cancer	5477 Patients	Urine and blood	Higher blood Cd and blood Pb	Men with elevated PSA had higher blood Cd and blood Pb than men with normal PSA	Wu et al. (2021) [39]
Se, Zn, Cu, Mg, Fe	Prostate cancer	50 Patients 50 Healthy controls	/Hair and nail	Significantly low levels of Se and Zn; high levels of Fe and Mg	Low levels of selenium and Zn and high levels of Cu, Fe and Mn appear to be associated with the risk of prostate cancer.	Karimi et al. (2012) [61]
Cd, Zn, As, Pb	Prostate cancer	25 Smokers 21 Non-smokers	Tissue	Higher Cd and lower Zn in smokers patients	Variations in metal levels can be drivers of disease progression	Neslund-Dudas et al. (2014) [61]

### Gastric cancer

The levels of Zn, Cr, Mn, Sn, Cu, Pb, Al, and Fe were investigated in 50 esophagus and gastric cancer tissue samples in a study by Sohrabi et al., with these findings being compared to that of healthy tissues. The samples were taken from 13 patients with esophageal cancer and 37 patients with gastric cancer. Significant differences in Zn, Cr, and Sn were observed between cancer and healthy tissues. Moreover, the researchers observed differences in the levels of some trace elements and heavy metals between genders, a finding which necessities further consideration in future investigations [76] (Table 4). In addition, Wang et al. observed a relationship between the concentration of heavy metals and human epidermal growth factor receptor type 2 (HER2) gene amplification in 105 gastric cancer patients. In this study, the concentration of As, Cr, Cu, Hg, Mn, Sb, Se, Pb, Sr, Tl, V, Sn and Zn significantly differed between gastric cancer patients and the 62 healthy controls. In addition, it was observed that Hg, Se, and Tl levels were noticeably increased in the HER2 positive group when compared to the HER2 negative group [77]. Feng et al. have detected the concentrations of 17 metals in gastric tissues, which have been associated with investigated rDNA copy number in gastric cancers and its link with existing biomarkers. This study was performed on paired tumor and adjacent normal tissues obtained from 65 gastric cancer patients, revealing high concentrations of As, Cd, Cr, Cu and Fe in both pathological and adjacent healthy tissues [78]. Moreover, rDNA copy number variation was related with the concentrations of certain metals and may be associated with metal exposure [78]. Fonseca-Nunes e al. have conducted a study case-control in the multicentric European Prospective Investigation into Cancer and Nutrition (EPIC) study, that included 456 primary incident gastric adenocarcinoma cases and 900 controls during 11 years of follow-up. They showed a decreased risk of gastric cancer related to higher body iron stores as measured by serum iron and ferritin [79].

**Table 4:** Heavy metals and gastric cancer

Metals	Cancer site	Sample size	Material	Level of metals	Impact	References
Zn, Cr, Mn, Sn, Cu, Pb, Al, Fe	Gastric and oesophagus cancer	After “50 patients 13 esophageal cancers 37 gastric cancers	Oesophagus and gastric cancer tissue	Significant differences in Zn, Cr, Mg, and Cu compared with healthy tissue. Cr level is higher in cancerous tissues; Cu level was significantly lowered in cancerous tissues; content of Zn was lower in esophageal cancer tissues; concentration of Sn in gastric cancer tissues was higher than the normal tissues; Fe in cancerous tissues was higher, Pb and Al were higher in esophageal cancer compared to the normal tissues	This difference may reflect the underlying mechanism of cellular changing during the tumorigenesis or direct exposure of these elements.	Sohrabi et al. (2021) [62]
As, Cr, Cu, Hg, Mn, Pb, Sb, Se, Sn, Sr, Ti, V, Zn	Gastric cancer	105 Patients 62 healthy controls	Plasma	As, Cr, Cu, Hg, Mn, Pb, Sb, Se, Sn, St, Ti, V, and Zn significantly different between gastric cancer patients and healthy controls.	Relationship between the concentration of heavy metals and human epidermal growth factor receptor type 2 (HER2) gene amplification. Mercury, selenium, and thallium levels were increased in the HER2 positive group	Wang et al. (2022) [63]
Ag, Al, As, Cd, Co, Cr, Cu, Fe, Mn, Mo, Ni, Pb, Sb, Se, Sr, V and Zn.	Gastric cancer	65 Patients	Tissue	High concentrations of As, Cd, Cr, Cu and Fe	rDNA copy number variation may be associated with metal exposure	Feng et al. (2020) [64]
Fe	Gastric cancer	456 Patients 900 healthy controls	Serum	High levels of Fe in patients vs. healthy controls	Decreased risk of gastric cancer related to iron body stores	Fonseca-Nunes e al. (2015) [65]

## Materials and methods

PubMed, Web of Science, and Scopus were systematically searched to identify relevant literature published up until the end of 2022 on the presence of heavy metals in biological samples of tumor patients. The search strategy was formulated according to the PECO guideline[80]. The key search terms used in the search strategy included “heavy metals” or “trace elements” combined with “cancer” or “tumor” or “carcinoma”. Additionally, all results reported in previous reviews and relevant meta-analyses were searched.

### Study selection and data collection

Identified studies were only included if they were compliant with the following standards: (1) the population (P) was restricted to patients with a confirmed cancer diagnosis; (2) exposure (E) to heavy metals, evidenced in biological samples; (3) the comparator (C) was specified in the research, including individuals without cancer; (4) the outcome (O) was cancer prevalence; (5) only studies involving human participants were included; and (6) the studies that were available in the English language. In some instances, this study incorporated research that did not present original data, such as review articles, if deemed relevant to the examined topics. However, the primary focus was on original works. Conversely, studies that did not assess heavy metals in biological samples were excluded. Subsequently, data were collected and categorized based on the malignancy of the patients.

## Conclusions

Examination of the literature available on the topic of metal-induced carcinogenesis indicates that there is enough substantial data to support the hypothesis of a direct relationship between the presence of heavy metals within the tissues and the increased risk of tumorigenesis. The levels and the type of metal involved in such processes differ substantially amongst lung, breast, prostate and gastric cancer patients; however, there is a collective agreement amongst the current literature that the levels of heavy metals are augmented in biological samples of tumor patients. However, although several mechanisms for this relationship were proposed, such as genotoxicity, oxidative stress, inhibition of DNA repair, just to quote some, there are current knowledge gaps within the literature that call for further research.

Data collected during this review work indicate the number of papers dealing with metals determined directly in cancer tissues (biopsies) is still rather limited, while this should be one of the main points to be addressed while trying to unveil the relation between heavy metals and cancer. We encourage researchers to explore this field in order to help understand such delicate aspect.

Moreover, it must be said it is rather difficult to compare data relative to different metals involved in the carcinogenesis of a given organ when their concentrations are measured in different biological samples (e.g. biopsies, adjacent normal tissues, plasma, urine, hair, nail, etc.), as the accumulation of metals can normally vary from tissue to tissue. Even the same metal can be unevenly distributed in different parts of the body. The significance of a study, in our opinion, could be increased when metals are determined in various biological samples, and not only in one. This could add soundness to the conclusion drawn from the collection of these data.

What is important, however, is that the awareness on the relationship between heavy metals and increased cancer risk, and the relative consequences for human health has been raised, and that significant counter-measures can be undertaken in order to reduce human exposure to these pollutants both in the environment and in the working place. Moreover, effective therapies should be available for those who have been exposed to chronic and acute contamination, although being the latter normally less dangerous, especially through detoxification of the organism with selective chelating agents or analogous treatments.

In conclusion, the presence of heavy metals in biological samples from tumor patients represents a critical area of study that requires ongoing research and a multidisciplinary approach to better understand the intricate relationships between heavy metal exposure and cancer. These insights hold the promise of improving our understanding of cancer etiology and potentially influencing public health measures to mitigate heavy metal exposure and reduce cancer risk.

As we continue to explore the complex network of connections between heavy metals and cancer, we hope that these discoveries will ultimately lead to more effective preventive measures, earlier detection, and improved treatment strategies for individuals affected by these devastating diseases. The collaborative efforts of researchers, clinicians, and policymakers are essential in addressing this urgent issue and enhancing the overall well-being of cancer patients and the general population.

## Data Availability

Not applicable.
